# Ultrasensitive DNA detection based on target-triggered rolling circle amplification and fluorescent poly(thymine)-templated copper nanoparticles†

**DOI:** 10.1039/c7ra11071e

**Published:** 2018-01-16

**Authors:** Kwan Woo Park, Chang Yeol Lee, Bhagwan S. Batule, Ki Soo Park, Hyun Gyu Park

**Affiliations:** Department of Chemical and Biomolecular Engineering (BK 21+ Program), KAIST Daehak-ro 291, Yuseong-gu Daejeon 34141 Republic of Korea hgpark@kaist.ac.kr +82-42-350-3910 +82-42-350-3932; Department of Biological Engineering, College of Engineering, Konkuk University Seoul 05029 Republic of Korea kskonkuk@gmail.com +82-2-450-3742 +82-2-450-3742

## Abstract

We describe a novel strategy for the ultrasensitive detection of target DNA based on rolling circle amplification (RCA) coupled with fluorescent poly(thymine)-templated copper nanoparticles (poly T-CuNPs). In the presence of target DNA, a padlock DNA probe that consists of two regions: a target DNA-specific region and a poly(adenine) region, is circularized by the ligation reaction, and the subsequent RCA reaction is promoted to generate long, concatemeric, single-stranded DNA (ssDNA) with a lot of repetitive poly T sequences. As a result, a large number of poly T-CuNPs are formed, exhibiting a highly fluorescent signal. However, in the absence of target DNA or in the presence of non-specific target DNA, the padlock DNA probe is not circularized and the subsequent RCA is not executed, leading to no production of fluorescent poly T-CuNPs. With this simple strategy, we successfully analyzed the target DNA with the ultralow detection limit of 7.79 aM, a value that is 3 or 7 orders of magnitude lower than those of previous RCA-based fluorescent DNA detection strategies. In addition, the developed system was demonstrated to selectively discriminate non-specific target DNAs with one-base mismatch, suggesting potential application in the accurate diagnosis of single nucleotide polymorphisms or mutations.

## Abstract 2

An ultrasensitive DNA detection method is developed based on target-triggered rolling circle amplification coupled with fluorescent poly(thymine)-templated copper nanoparticles.

## Graphical abstract

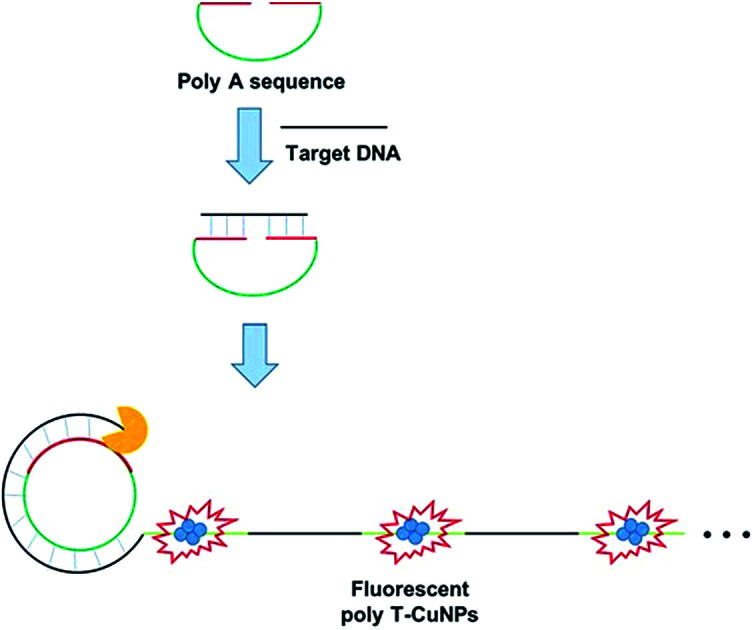

## Introduction

Nucleic acid detection is essential in the area of bioanalytical research including clinical diagnostics, biomedicine, and forensics since all organisms have their own unique genetic information, which could be a promising identification marker.^1–4^ However, the amount of target nucleic acids in biological samples is too low to be reliably analyzed and thus amplification techniques are inevitably required. Until now, polymerase chain reaction (PCR) has been widely utilized to detect the low-abundance target nucleic acids,^5–8^ but it has critical drawbacks including the requirement of thermocycling, technical expertise, and expensive instrumentation, which are not suitable for point-of-care-testing in facility-limited settings.^9–11^

Recently, various isothermal nucleic acid amplification methods have been reported as promising alternatives to replace PCR-based assays.^12–15^ Notably, rolling circle amplification (RCA) has received the special attention due to its unique feature to produce a long, concatemeric, single-stranded DNA (ssDNA) at the constant temperature.^16,17^ In addition, various signaling methods for the effective analysis of RCA products have been suggested, which includes colorimetry,^18,19^ electrochemistry,^20,21^ and fluorometry.^17,22–24^ Among these, the fluorescent approaches have been thoroughly studied due to their reliability, simplicity, and sensitivity. The examples in this type of signaling strategies utilize fluorescent dye-labeled dNTPs,^22^ dye-labeled DNA probe,^24^ molecular beacon,^17^ fluorescence resonance energy transfer (FRET) probe,^25^ and intercalating dyes such as SYBR green.^23^ Although each method provides the promising results for the effective analysis of RCA products, these strategies have several limitations. For example, the dye-labeled dNTPs significantly reduce the amplification efficiency of RCA and the dye-labeled probes increase the overall assay cost. In addition, SYBR green dye that can be utilized in the real-time monitoring of RCA, inherently accompanies the high background signal due to its nonspecific intercalation property, which significantly reduces the detection sensitivity. Therefore, a great incentive still exists for the development of sensitive and cost-effective methods for the target nucleic acids based on the fluorescence monitoring of RCA reaction.

Towards this goal, we developed a simple, label-free, and ultrasensitive DNA detection strategy, which relies on target-triggered RCA and fluorescent poly(thymine)-templated copper nanoparticles (poly T-CuNPs), a compelling alternative to conventional, organic fluorescent dyes due to their outstanding spectroscopic and photophysical properties, low toxicity, and biocompatibility.^26^ Notably, poly T-CuNPs are known to be more sensitive and quickly prepared as compared to DNA-templated silver nanoclusters (DNA-AgNCs), a well-known fluorescent signal reporter for the nucleic acid detection.^27–31^ In principle, the presence of target DNA induces the circularization of a padlock DNA probe composed of a target DNA-specific region and a poly(adenine) region, and thus the subsequent RCA is promoted to generate a long, concatemeric ssDNA with a lot of repetitive poly T sequences. As a result, a large number of highly fluorescent poly T-CuNPs are produced. Based on this simple strategy, the target DNA that encodes microRNA-141 (miR-141), a promising marker for human prostate cancer, was successfully analyzed with the ultra-high sensitivity and selectivity.^32,33^

## Experimental

### Materials

All DNA sequences (Table S1†) were synthesized from GenoTech (Daejeon, Korea) and purified by the standard desalting except 5′-end phosphorylated padlock DNA probe which was purified by polyacrylamide gel electrophoresis (PAGE). T4 DNA ligase, phi29 DNA polymerase, bovine serum albumin (BSA), and dNTPs were purchased from New England Biolabs (Beverly, MA, USA). Copper sulfate, sodium ascorbate, 3-(*N*-morpholino) propanesulfonic acid (MOPS), and sodium chloride were purchased from Sigma-Aldrich (St. Louis, MO, USA). All chemicals were of analytical grade and used without further purification.

### Preparation of circularized DNA probe

The ligation solution (19 μL) composed of 1 μM of padlock DNA probe, 1× T4 DNA ligase reaction buffer (50 mM Tris–HCl, 10 mM MgCl_2_, 1 mM ATP, 10 mM DTT, pH 7.5), and target DNA at varying concentrations or 10 pM of non-specific target DNAs was first heated at 95 °C for 5 min, and then cooled slowly to 16 °C for 15 min to form a target DNA/padlock DNA probe complex. To this solution, 10 U of T4 DNA ligase (1 μL) was added, which was incubated for 60 min to make the circularized DNA probe. Finally, the ligation reaction was stopped by heating the solution at 65 °C for 10 min and the target DNA/circularized DNA probe complex was stored at 4 °C for further use.

### Target DNA-triggered RCA reaction

The RCA reaction solution (40 μL) composed of as-prepared target DNA/circularized DNA probe complex, 500 μM of dNTPs, 4 μg of BSA, 1× phi29 DNA polymerase buffer (50 mM Tris–HCl, 10 mM MgCl_2_, 10 mM (NH_4_)_2_SO_4_, 4 mM DTT, pH 7.5), and 10 U of phi29 DNA polymerase was incubated at 30 °C for 30 min and then terminated by heating the solution at 65 °C for 10 min. The resulting product was stored at 4 °C for further use.

### Synthesis of poly T-CuNPs

After the target-triggered RCA reaction, the resulting product was mixed with 2 mM of sodium ascorbate and 0.5 mM of CuSO_4_ in MOPS buffer (10 mM MOPS, 150 mM NaCl, pH 7.6), which was then incubated at room temperature in the dark for 5 min with gentle shaking to produce the poly T-CuNPs. The as-prepared poly T-CuNPs were characterized by TEM analysis (Fig. S1†) and their fluorescence intensities were measured at the excitation wavelength of 340 nm.

### Detection of target DNAs in human serum (1%)

The target DNA at varying concentrations and non-specific target DNAs with one-base mismatch (T1, T2, and T3) were spiked into the diluted human serum, which were subsequently analyzed using the same procedure to detect the target DNA in the buffer solution (target DNA-triggered RCA reaction and synthesis of poly T-CuNPs).

### Instrumentation

The gel image was obtained by using Bio-Rad Gel Doc™ Ez Imager (Hercules, CA, USA) and the fluorescence intensities were measured using a Tecan Infinite M200 pro microplate reader (Mannedorf, Switzerland) and black, 384-well Greiner Bio-One microplates (ref: 781077). The TEM image of as-prepared poly T-CuNPs was taken by using a field-emission transmission electron microscopy (Tecnai, FEI, Netherlands) operating at an accelerating voltage of 300 kV.

## Results and discussion

**Fig. 1:**
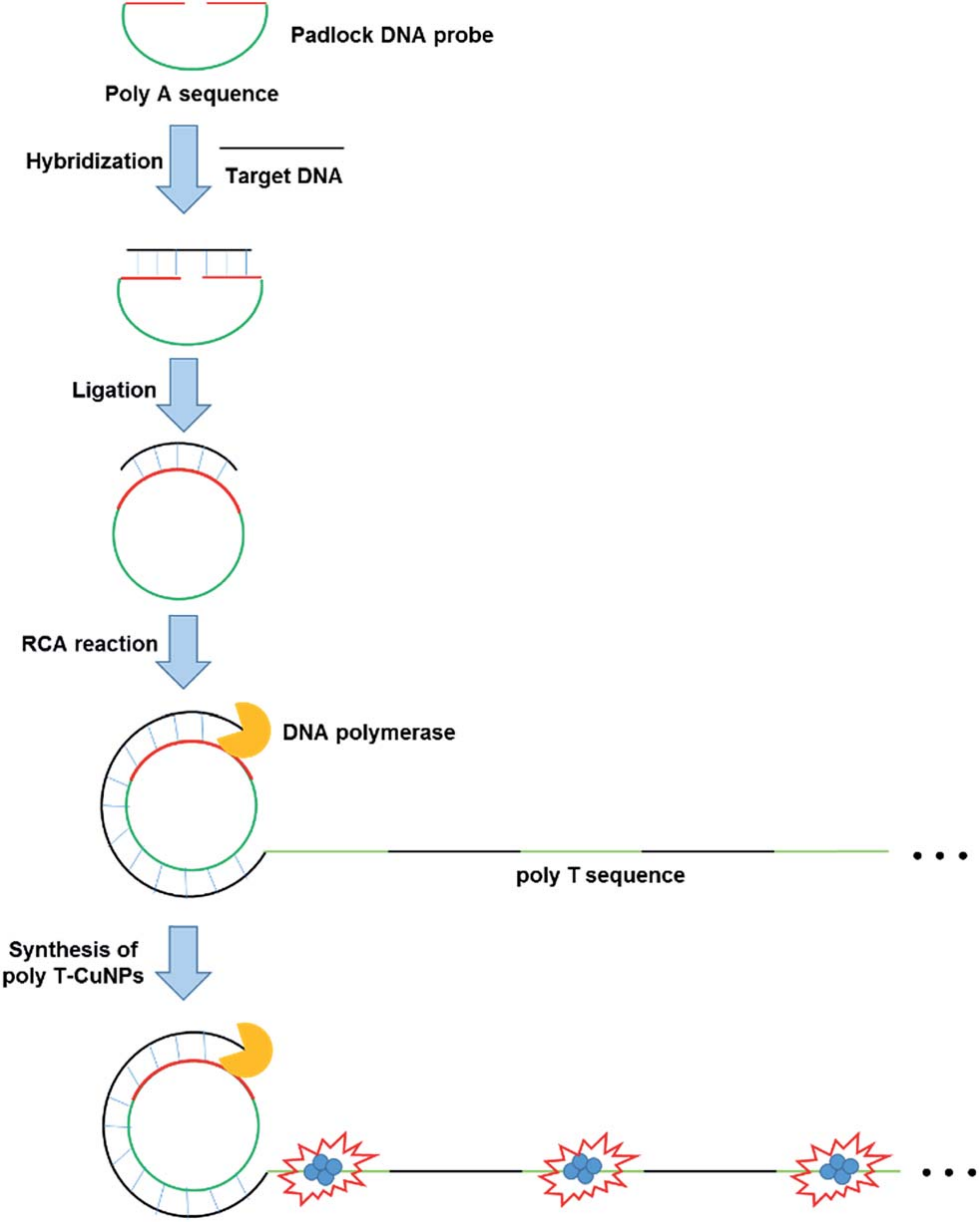
Schematic illustration of the ultrasensitive DNA detection strategy based on the target-triggered RCA coupled with the fluorescent poly T-CuNPs.

The overall procedure for the ultrasensitive DNA detection based on the target-triggered RCA coupled with the fluorescent poly T-CuNPs is schematically depicted in Fig. 1. In this strategy, the padlock DNA probe which consists of two regions: one is complementary to target DNA and the other is 30-mer of poly(adenine) sequence, is utilized as a key detection component. In the presence of target DNA, the padlock DNA probe forms the target DNA/padlock DNA probe complex to be circularized by T4 DNA ligase. The RCA reaction is then promoted by phi29 DNA polymerase, leading to the generation of the long, concatemeric ssDNA. Importantly, the resulting RCA product contains a lot of repetitive poly T sequences, which consequently leads to the production of highly fluorescent poly T-CuNPs. On the other hand, when target DNA is absent or non-specific target DNA is present, the padlock DNA probe is not circularized to perform the RCA reaction and thus fluorescent poly T-CuNPs are not formed, which is manifested by the negligible fluorescence signal. With this simple working principle, the target DNA is specifically detected with the ultrahigh sensitivity.

**Fig. 2:**
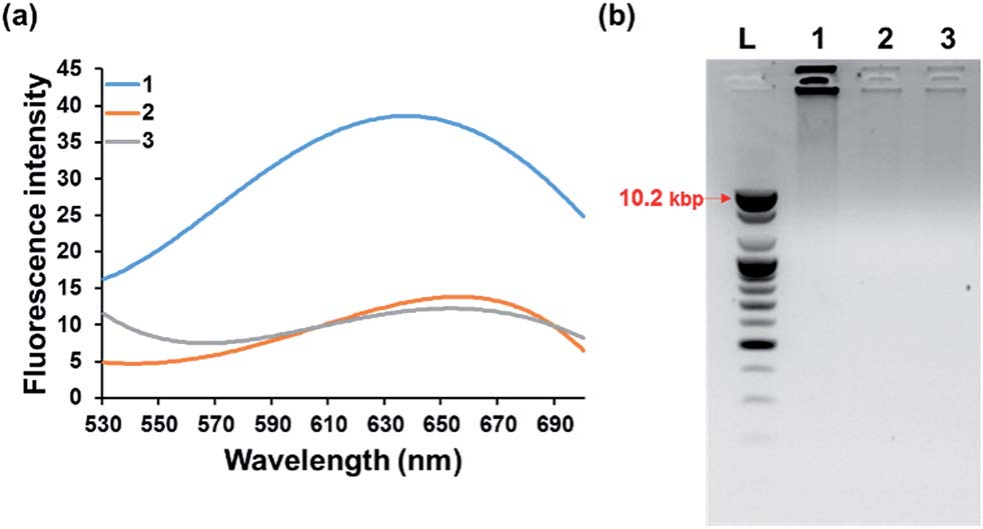
Detection feasibility of the developed strategy. (a) Fluorescence emission spectra after the target-triggered RCA and subsequent synthesis of poly T-CuNPs. (b) Gel electrophoresis image of the reaction products after the target-triggered RCA. L: 1 kb DNA ladder, 1: padlock DNA probe + target DNA, 2: padlock DNA probe, and 3: target DNA. The final concentrations of padlock DNA probe and target DNA are 100 nM and 1 nM, respectively.

We first confirmed the detection feasibility of the proposed strategy by measuring the fluorescence emission intensities from poly T-CuNPs. As envisioned in the design of the new strategy, the high fluorescence signal from poly T-CuNPs with the emission maximum at 650 nm upon the excitation at 340 nm was observed in the presence of both padlock DNA probe and target DNA (1, Fig. 2(a)). In contrast, the weak fluorescence signal was obtained when either of the two components (padlock DNA probe and target DNA) was not present (2 and 3, Fig. 2(a)). To further support the fluorescence results in Fig. 2(a), the gel electrophoresis analysis of products obtained after the target-triggered RCA reaction was executed. As shown in Fig. 2(b), the RCA products with high molecular weights were obtained near the loading well (>10.2 kbp) only in the presence of both padlock DNA probe and target DNA (lane 1, Fig. 2(b)), which was not observed in the negative controls where either the padlock DNA probe or target DNA was not included (lanes 2 and 3, Fig. 2(b)). Overall, the results obtained from both fluorescence measurement and gel electrophoresis were well matched, which clearly verifies the detection feasibility of the proposed method.

Next, we optimized the reaction conditions including the RCA reaction time and the concentration of Cu^2+^ required for the ultrasensitive detection of target DNA. The RCA reaction time was first optimized by measuring the fluorescence signals of a single-stranded DNA specific fluorescent dye, SYBR green II in a real-time signal acquisition mode.^34^ The results in Fig. S2† show that the fluorescence signals remained quite low in the absence of target DNA, but it gradually increased to reach a plateau after 30 min in the presence of target DNA. In addition, we optimized the concentration of Cu^2+^ for the effective synthesis of fluorescent CuNPs since dithiothreitol (DTT) essential for the RCA reaction can scavenge on Cu^2+^ through its complexation with sulfhydryl groups in DTT.^35^ As shown in Fig. S3,† the highest signal-to-noise ratio was obtained from 0.5 mM of Cu^2+^. Therefore, we selected 30 min of RCA reaction and 0.5 mM of Cu^2+^ for further experiments.

**Fig. 3:**
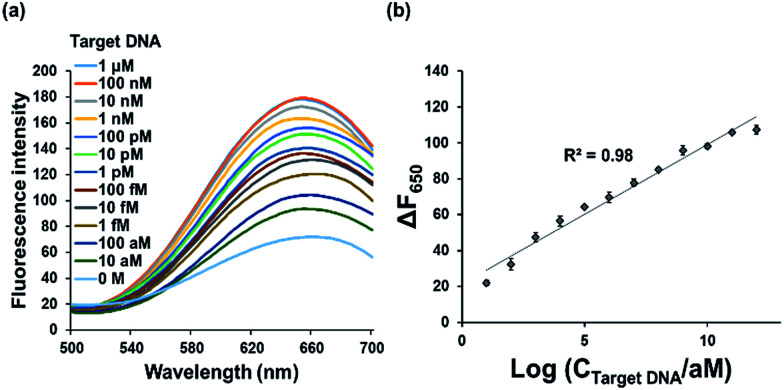
Detection sensitivity of the developed strategy. (a) Fluorescence emission spectra and (b) Δ*F*_650_ (*F* − *F*_0_, where *F*_0_ and *F* are the fluorescence intensities at 650 nm in the absence and presence of target DNA, respectively) after the target-triggered RCA and subsequent synthesis of poly T-CuNPs in the presence of varying concentrations of target DNA. The line in (b) indicates the linear relationship between Δ*F*_650_ and logarithm of target DNA concentration. The final concentration of padlock DNA probe is 1 μM. All the experiments were performed in triplicate.

Under the optimized reaction conditions, the detection sensitivity of our strategy was assessed by measuring the fluorescence intensities at 650 nm at varying concentrations of target DNA. As shown in Fig. 3, the fluorescence intensities increased with increasing concentrations of target DNA and an excellent linear relationship between Δ*F*_650_ and logarithm of target DNA concentration was found in the range from 10 aM to 1 μM (*R*^2^ = 0.98) with the limit of detection of *ca.* 7.79 aM (3*σ*/slope), a value that is significantly lower or slightly higher than those from other RCA-based fluorescent biosensors to detect the target DNA.^25,36,37^ The ultra-high sensitivity of present method is attributed to the synergistic effect of RCA and highly fluorescent poly T-CuNPs. Importantly, it should be emphasized that this method is more sensitive than RCA-based one that relies on DNA-AgNCs, which implies that CuNPs-based fluorescent signaling is more effective than AgNCs.^27^

**Fig. 4:**
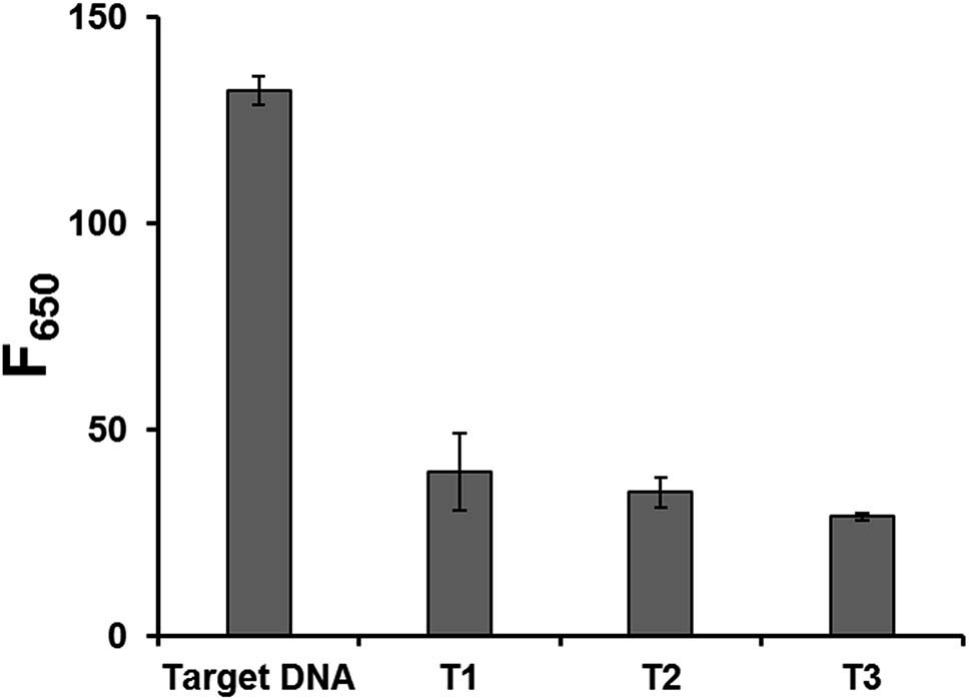
Detection selectivity of the developed strategy. Fluorescence intensities at 650 nm (*F*_650_) in the presence of target DNA and non-specific target DNAs with one base mismatch (T1, T2, and T3) (Table S1†). The final concentrations of padlock DNA probe, target DNA, and non-specific target DNAs are 1 μM, 10 pM, and 10 pM, respectively. All the experiments were performed in triplicate.

The detection specificity of our system was also evaluated by testing non-specific target DNAs with one base mismatch. The results in Fig. 4 show that only the target DNA led to the significantly increased fluorescence while the non-specific target DNAs with one base mismatch resulted in the negligible fluorescence. These results clearly demonstrate that this system is highly selective, suggesting the potential application for the simple and sensitive SNP genotyping or mutation detection.^38–42^

In addition, the practical applicability of the developed method was demonstrated by analyzing the mock clinical samples in which the target DNAs were spiked in human serum.^43–46^ As shown in Fig. S4,† the target DNAs with varying concentrations were successfully analyzed in human serum with the high selectivity, confirming its potential for the detection of target DNAs in real, clinical samples.

## Conclusions

In this study, we developed an ultrasensitive DNA detection strategy based on target-triggered RCA coupled with the fluorescent poly T-CuNPs. By taking advantage of the high amplification capability of RCA and highly emissive fluorescence of poly T-CuNPs, we successfully detected the target DNA that encodes miR-141 with the high sensitivity and selectivity, which does not require any expensive labeling and complicated assay procedures. In addition, the system can be expanded to the detection of long target DNAs by employing an additional primer that initiates RCA (Fig. S5†). Importantly, this method gave the limit of detection of 7.79 aM, a value that is 3 or 7 orders of magnitude lower than those of previous RCA-based fluorescent DNA detection strategies. Moreover, the target DNA was successfully discriminated from non-specific target DNAs only with one-base mismatch, which suggests the future application of the proposed method for SNPs genotyping or mutation detection. We strongly believe that our strategy can be a powerful tool for the early diagnosis of specific nucleic acids related to infectious diseases or cancers.

## Conflicts of interest

There are no conflicts to declare.

## Supplementary Material

RA-008-C7RA11071E-s001
