# Supplementary material for: Ultrasensitive DNA detection based on target-triggered rolling circle amplification and fluorescent poly(thymine)-templated copper nanoparticles
Source: RSC Adv. 2018 Jan 16;8(4):1958–62. doi: 10.1039/c7ra11071e (PMC9077274; doi:10.1039/c7ra11071e)
Supplement: RA-008-C7RA11071E-s001 [file RA-008-C7RA11071E-s001.pdf]

Electronic Supplementary Material (ESI) for RSC Advances.
This journal is © The Royal Society of Chemistry 2017

Supporting Information for

Ultrasensitive DNA detection based on target-triggered rolling circle amplification and fluorescent poly (thymine)-templated copper nanoparticles

By Kwan Woo Park,[†a] Chang Yeol Lee,[†a] Bhagwan S. Batule,[a] Ki Soo Park*[b] and Hyun Gyu Park*[a]

[a]Department of Chemical and Biomolecular Engineering (BK 21+ program),

KAIST, Daehak-ro 291, Yuseong-gu, Daejeon 34141, Republic of Korea

[b]Department of Biological Engineering, College of Engineering,

Konkuk University, Seoul 05029, Republic of Korea

[†] The authors equally contributed to this work.

* To whom correspondence should be addressed.

E-mail: hgpark@kaist.ac.kr (H.G. Park); Phone: +82-42-350-3932; Fax: +82-42-350-3910.

E-mail: kskonkuk@gmail.com (K.S. Park); Phone: +82-2-450-3742; Fax: +82-2-450-3742.

Table S1 DNA sequences utilized in this study.

Name	DNA sequence (5' → 3')
Padlock DNA probe(a)	P–AGA CAG TGT TAA AAA AAA AAA AAA AAA AAA AAA AAA AAA AAC CAT CTT TAC C
Target DNA	TAA CAC TGT CTG GTA AAG ATG G
T1(b)	TAA CAC TGT CTA GTA AAG ATG G
T2(b)	TAA CAC TGT CTC GTA AAG ATG G
T3(b)	TAA CAC TGT CTT GTA AAG ATG G

(a) 'P' indicates the phosphorylation.

(b) One base mismatch site in T1, T2, and T3 is underlined.

Fig. S1 TEM image of the as-prepared poly T-CuNPs.

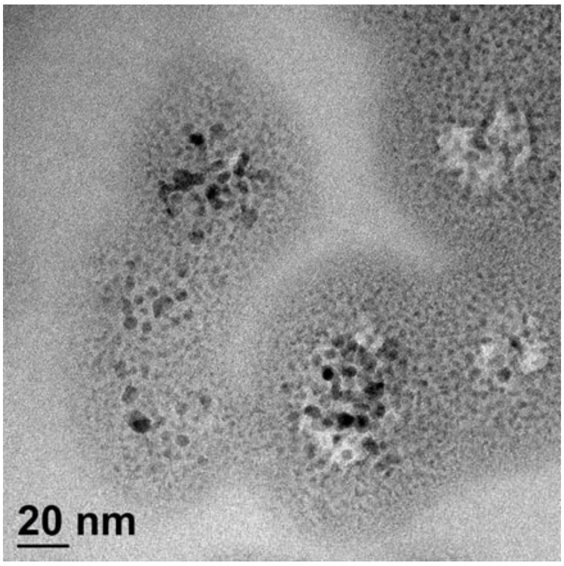

The as-prepared poly T-CuNPs are spherical in shape and have diameters of approximately 3 nm, which is in accordance with the previous report.[1]

Fig. S2 Time-dependent fluorescence intensities at 525 nm from SYBR green II during the target-triggered RCA. The RCA reaction was executed at 30 °C and the fluorescence intensities were monitored at every 1 min using C1000™ thermal cycler (Bio-Rad, CA, USA). The final concentrations of padlock DNA probe and target DNA are 100 nM and 1 nM, respectively.

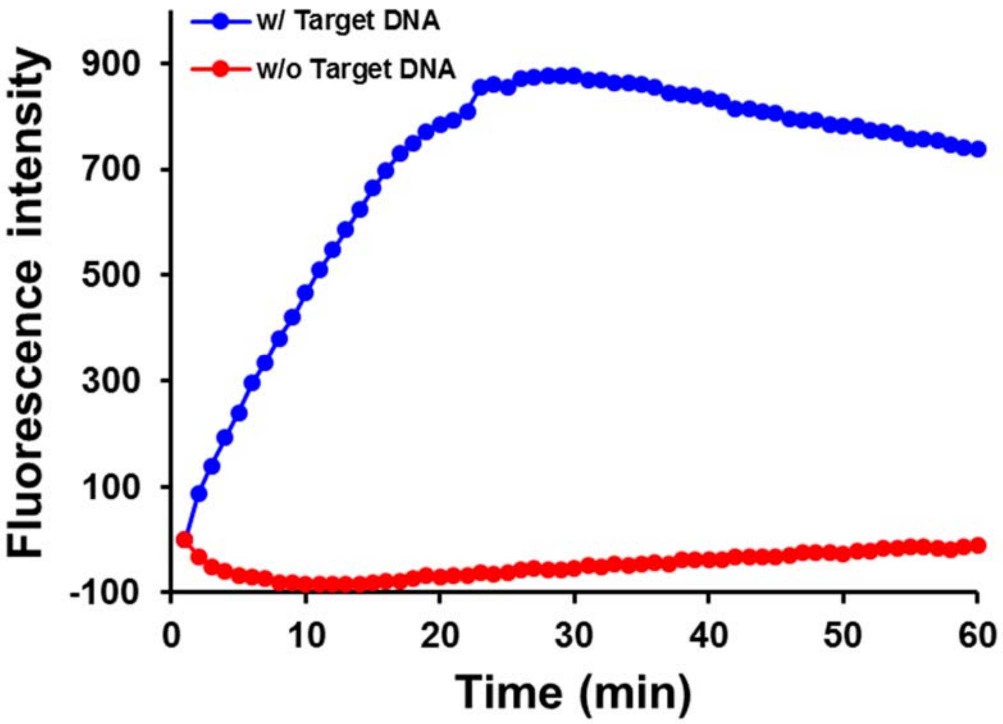

Fig. S3 Optimization of the Cu^{2+} concentration for the efficient analysis of target DNA. The signal-to-noise ratio (S/N) is defined as F/F_0, where F_0 and F are the fluorescence intensities at 650 nm from poly T-CuNPs synthesized after the target-triggered RCA in the absence and presence of target DNA, respectively. The final concentrations of padlock DNA probe and target DNA are 100 nM and 1 nM, respectively.

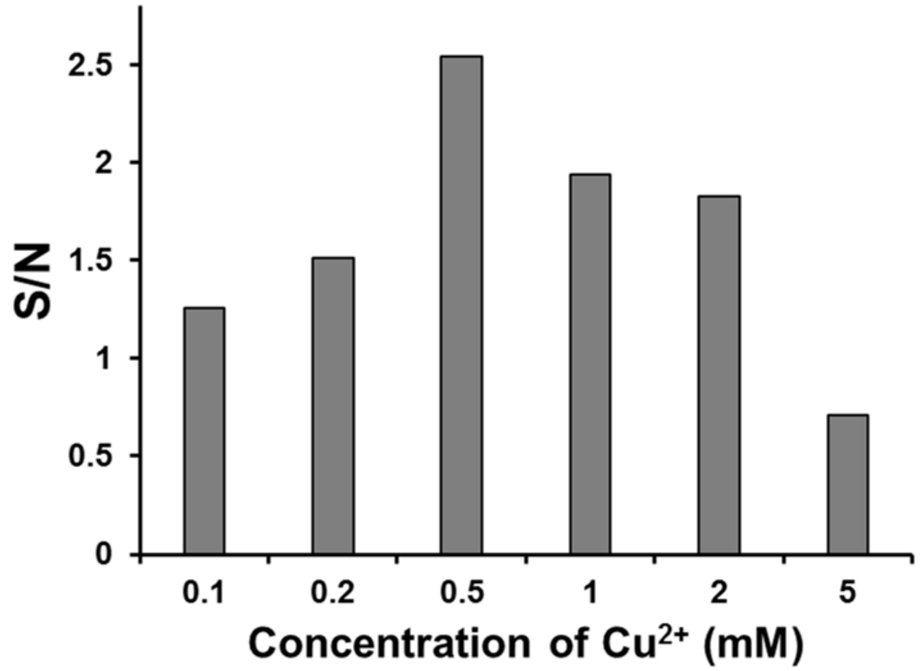

Fig. S4 Practical applicability of the developed strategy. ΔF_{650} ($F-F_0$, where F_0 and F are the fluorescence intensities at 650 nm in the absence and presence of target DNA, respectively) in the presence of varying concentrations of target DNA and non-specific target DNAs with one base mismatch (10 nM, Table S1‡) in human serum. The final concentration of padlock DNA probe is 1 µM. All the experiments were performed in triplicate.

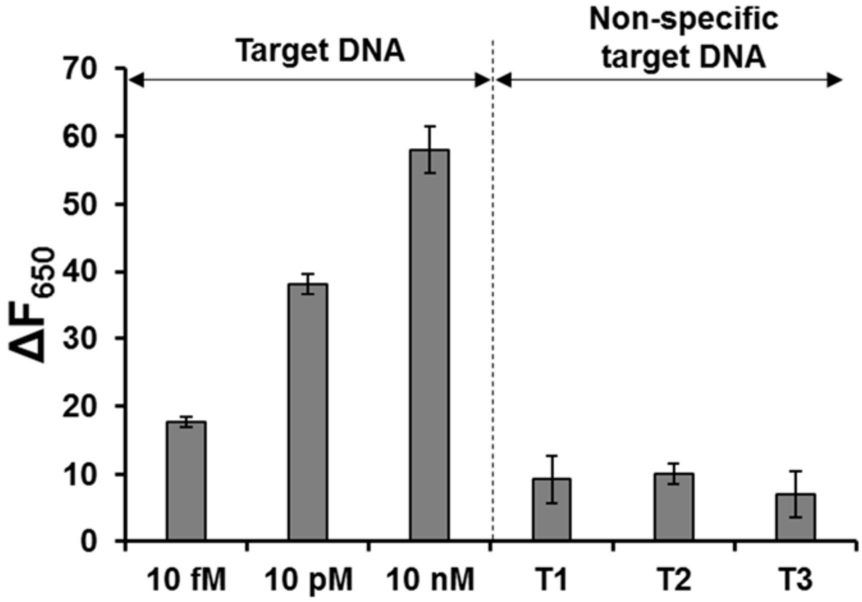

Fig. S5 Schematic illustration for the detection of long target DNAs.

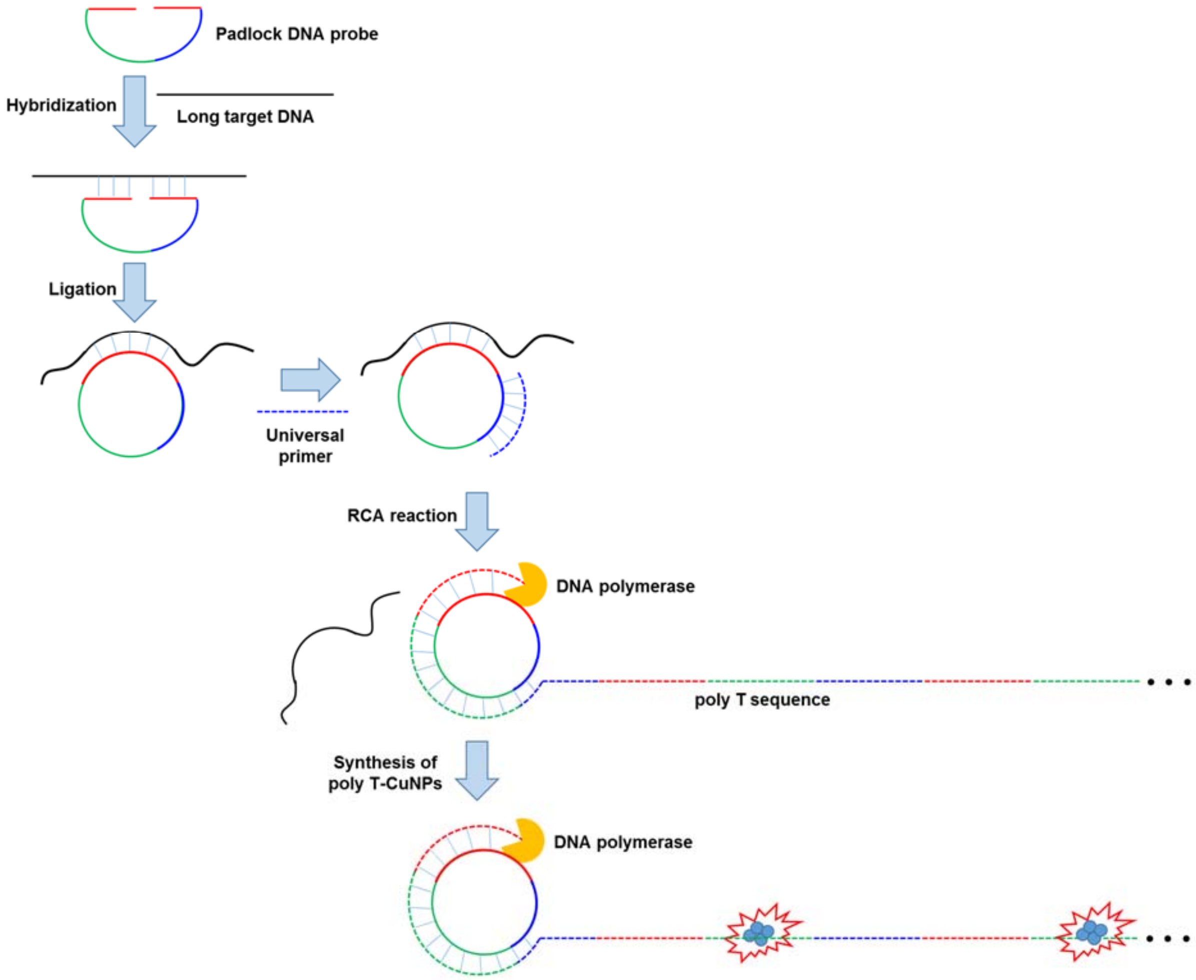

References

1 F. Peng, Z. Liu, W. Li, Y. Huang, Z. Nie and S. Yao, *Anal. Met.*, 2015, **7**, 4355-4361.
